# Supplementary material for: AMBRA1 p.Gln30Arg Mutation, Identified in a Cowden Syndrome Family, Exhibits Hyperproliferative Potential in hTERT-RPE1 Cells
Source: Int J Mol Sci. 2022 Sep 22;23(19):11124. doi: 10.3390/ijms231911124 (PMC9570079; doi:10.3390/ijms231911124)
Supplement: Supplementary file 1 [file ijms-23-11124-s001.zip › ijms-1889813-supplementary.pdf]

Supplementary Materials

Whole exome sequencing

Genomic DNA was isolated from the peripheral blood lymphocytes of both affected and unaffected members of the CS family, using the QIAamp DNA mini kit (QIAgen, Hilden, Germany). Sequencing libraries were prepared using the Truseq Exome library prep kit following the manufacturer's protocol (Illumina Inc., San Diego, CA, USA), and then they were sequenced using the MiSeq platform with a 350 and a 250 bp paired-end module and estimated with 100x coverage (Illumina, San Diego, CA, USA).

Briefly, the bioinformatics pipeline for germline variant calling involved the following: trimming the Illumina adapter and low-quality sequences using Trimmomatic (v.0.32) software; mapping the trimmed reads on human reference genome version hg19/GRCh37 using Burrows-Wheeler Aligner (BWA) (v.0.7.17); sorting the aligned reads using SAMtools (v. 0.1.19); removal of duplicate reads using Picard (v. 2.21.1); and, finally, calling the germline variants using GATK's HaplotypeCaller and VariantRecalibraor software. Functional annotation of the identified variants was implemented using ANNOVAR.

Prediction of AMBRA1 protein structural change using Alphafold2

The 3D structure analysis of the AMBRA1 missense variants was performed using Alphafold2, which is an efficient machine learning algorithm for protein structure prediction. We carried out the analysis using an advanced AlphaFold2 implementation from ColabFold [26], following the parameters recommended by Kabir et al., 2022 [27]. We ran the Alphafold2 program using 100 amino acids from the AMBRA1 N-terminal region comprising the Q30 variant site and WD1 domain, and 200 amino acids from the C-terminal region comprising the R1195 variant site and disordered region. We considered the pLDDT (Local Distance Difference Test) scores to evaluate the prediction confidence for the wildtype and variant sequences of both the Q30R and R1195S mutations. The prediction accuracy of the AlphaFold2 program for the wildtype and mutation residue of the AMBRA1 Q30R and R1195S mutations was estimated by the distribution of the pLDDT score per amino acid position, which was higher at the conserved AMBRA1 N-terminal region and lower at the disordered and less conserved AMBRA1 C-terminal region.

CRISPR/Cas9-mediated AMBRA1 Q30R editing in hTERT-RPE1 cells

hTERT-RPE1 cells were maintained in Dulbecco's Modified Eagle's Medium (DMEM)-low glucose supplemented with 10% heat-inactivated fetal bovine serum and 1% Penicillin–Streptomycin (Pen-Strep) and incubated at 37 °C and 5% CO2.

To knock out *AMBRA1* in hTERT-RPE1 cells, we designed a pair of guide RNAs targeted against intron 6 and exon 7 of the AMBRA1 genomic locus (Figure S1). Together with the CRISPR/Cas9 complex, these guide RNAs generate a deletion of approximately 1093 bp that could abolish AMBRA1 protein expression, as detected through Western blotting, as described later. For *AMBRA1* Q30R editing (c.A89G), we designed a guide RNA (Figure S1) targeting exon 2 and a single-stranded

oligodeoxynucleotide (ssODN) to facilitate homology-directed repair at the Cas9 cut site. The ssODN we designed carries homology arms of 60 bp with the targeted mutation at the center (A>G) (Figure S1), along with another nucleotide conversion (G>C) at 3′ immediately at the targeted mutation. Therefore, CAG>CGC editing helped us to create a Q30R mutation and to abolish the PAM site that prevented repetitive editing by the Cas9 enzyme. However, we represented the AMBRA1 Q30R mutants as $AMBRA1^{Q30R\ A/G}$ and $AMBRA1^{Q30R\ G/G}$, with A/G or G/G indicative of the actual A>G mutation observed in the patients for easier understanding. Additionally, the 5′ and 3′ ends of the ssODN were modified with phosphorothioate bonds to make it more stable inside the cell, as recommended by the manufacturer (Integrated DNA Technologies IDT, San Diego, CA, USA).

For the electroporation, we used a Neon transfection system (Invitrogen, Waltham, MA, USA). Briefly, 5.0×10^5 cells were harvested and resuspended in 40 µl of Resuspension Buffer R (Invitrogen, Waltham, MA, USA). The CRISPR/Cas9-RNP complex (20 µM of Cas9 enzyme and 20 µM of tracrRNA:crRNA) was added to the cell suspension, and for AMBRA1 Q30R editing, 35 µM of the ssODN was added in addition to the CRISPR/Cas9-RNP complex. Further, the cell suspension with RNP complexes was loaded onto 10 µl electroporation tips, and electroporation was performed at 1350 mV for 20 milliseconds and 2 pulses using a Neon Electroporator (Invitrogen, Waltham, MA, USA). After electroporation, cells were rescued in pre-warmed DMEM-low glucose medium and plated on a 6-well plate for proliferation. After 2 days, the cells were further subcultured in 10 cm dishes to isolate individual clones.

To confirm the *AMBRA1* knockout, genomic DNA was isolated from the clones, and we amplified the region spanning intron 6 and exon 7 of the guide RNAs to produce a fragment of 204bp if there was a deletion or 1300 bp if the genomic region was intact and resolved it in 10% PAGE (Figure S2A). Further, the AMBRA1 protein was detected using Western blotting (Figure S2B). To confirm *AMBRA1* Q30R editing, we amplified the exon 2 region and performed Sanger sequencing (Figure S2C).

Western Blotting

Cells were seeded in a 10 cm dish and starved for 24 h in Opti-MEM medium. Total protein was extracted from both the control and starved cells using RIPA buffer (Sigma Aldrich, St. Louis, MO, USA), and the protein concentration was quantified using a Qubit protein assay (Invitrogen, Waltham, MA, USA). An amount of 50 µg of protein was loaded into each well filled with 4-12% NuPAGE precast gel (Invitrogen, Waltham, MA, USA) and separated by sodium dodecyl sulphate-polyacrylamide gel electrophoresis (SDS-PAGE), followed by transfer to a low-fluorescent polyvinylidene fluoride (PVDF) membrane (Invitrogen, Waltham, MA, USA). The blot was blocked using the EveryBlot blocking reagent (Bio-Rad, Hercules, CA, USA) at room temperature for 10 min and incubated in mouse anti-AMBRA1 (#24907, CST, Danvers, MA, USA, 1:1000) or rabbit anti-cyclinD1 (#2978, CST, Danvers, MA, USA, 1:1000) primary antibody solution diluted in the EveryBlot blocking reagent at 4°C overnight. After washing the blots with

TBS-T buffer, the blots were then incubate. d with anti-mouse HRP secondary antibody (ab6820, Abcam, Cambridge, UK ,1:10000 in EveryBlot reagent) and anti-actin hFAB ™ Rhodamine (#12004163, Abcam, Cambridge, UK ,1:5000) for 1 h at room temperature. Subsequently, the membrane was washed thrice with TBS-T and twice with TBS buffer, and the expression of the protein was documented using a ChemiDoc Touch imaging system (Bio-Rad, Hercules, CA, USA).

Flow cytometry

The distribution of the cells in cell cycle phases was analyzed through flow cytometry. In brief, cells were seeded in a 10 cm dish and starved for 24 h in Opti-MEM medium. Both the control and starved cells were harvested and fixed in 70% chilled ethanol, followed by storage at -20°C overnight to enhance fixation. Cells were centrifuged for 5 min at 2000 rpm and washed with 5 ml FACS buffer (2% FBS, 1 mM EDTA in PBS) twice, followed by centrifugation at 2000 rpm for 5 min. After centrifugation, cells were resuspended in 100 µl FACS buffer containing mouse anti-Ki67 primary antibody (ab8191, Abcam, 1:100) and incubated at 37°C for 30 min. Cells were washed with 5 ml of FACS buffer followed by incubation with anti-mouse Alexa flour 488 secondary antibody (1:500 in FACS buffer) at 37°C for 30 min. Further, cells were washed with 5 ml of FACS buffer and incubated with 500 µl of propidium iodide solution (50 µg/ml RNaseA, 40 µg/ml Propidium Iodide, and 2 mM MgCl2 in PBS) at 37°C for 30 min, and then they proceeded to flow cytometry analysis. The stained cells were analyzed using a BD Accuri flow cytometer (BD Biosciences, USA) with 10,000 events per sample at a flow rate of 400 events/sec. Fractions of cells in different cell cycle phases were calculated using FCS express (De Novo Software, Los Angeles, CA, USA).

Immunocytochemistry

For immunocytochemistry, 1.0×10^5 cells were cultured in DMEM-low glucose medium and starved for 24 h in Gibco Opti-MEM medium (Invitrogen, Waltham, MA, USA). Both the control and starved cells were fixed in 3.7% formaldehyde for 10 min, washed thrice with PBS, and then permeabilized with 0.5% TritonX for 15 min. The permeabilized cells were then blocked with 1% BSA for 30 min at room temperature after washing thrice with PBS. Following blocking, cells were incubated with rabbit anti-arl13B primary antibody (C827D13, Proteintech, 1:500), the primary cilia marker, overnight at 4°C. Cells were then washed with PBS and incubated with Alexa-Fluor®-conjugated secondary antibody (Invitrogen, USA) for 1 h at room temperature. Cells were then washed thrice with PBS and mounted using Vectashield antifade medium containing DAPI (Vector Laboratories, USA). The immunostained cells were imaged under a Deltavision Personal DV fluorescent microscope (GE Healthcare, Chicago, IL, USA). Quantification analysis of the nucleus and primary cilia was performed using the Volocity 6.5 software (Quorum technologies, Pushlinch, ON, Canada).

Manipulation of ambra1 in zebrafish

All zebrafish experiments were carried out with permission from the Committee for Animal Care and Use of the National Institute of Genetics, Japan (NIG#29-14, approved on 04 July 2017). Adult zebrafish were

maintained on a 13 h light/11 h dark cycle. Embryos were kept at 28℃ until processing for phenotypic analysis.

In zebrafish, *ambra1* codes for two paralog nonredundant genes, ambra1a and ambra1b (14). To knock out ambra1a and b using CRISPR/Cas9, we used four sets of sgRNA sequences designed by Wu and colleagues [28]. sgRNA synthesis was performed as described [29]. We performed RNP complex formation as described [28]. We injected 1 nL of RNP solution into one-cell-stage embryos.

To knock down *ambra1a* and *ambra1b*, antisense morpholinos (MOs) were designed against the ATG translation initiation site according to the following sequences: MO1-ambra1a (5'-TCTGTCCCAGCTTCATCTCCAAACA-3') and MO1-ambra1b (5'-TTCTGTTCTGCACAGCCATTTTTCC-3') (Gene Tools, Philomath, OR, USA). The MOs were reconstituted in nuclease-free water with a final concentration of 1 ng/μl. We injected 1 nL of the MO solution into the one-cell-stage embryos. Phenotypic analysis was performed using a Leica M165 FC microscope (Wetzlar, Germany).

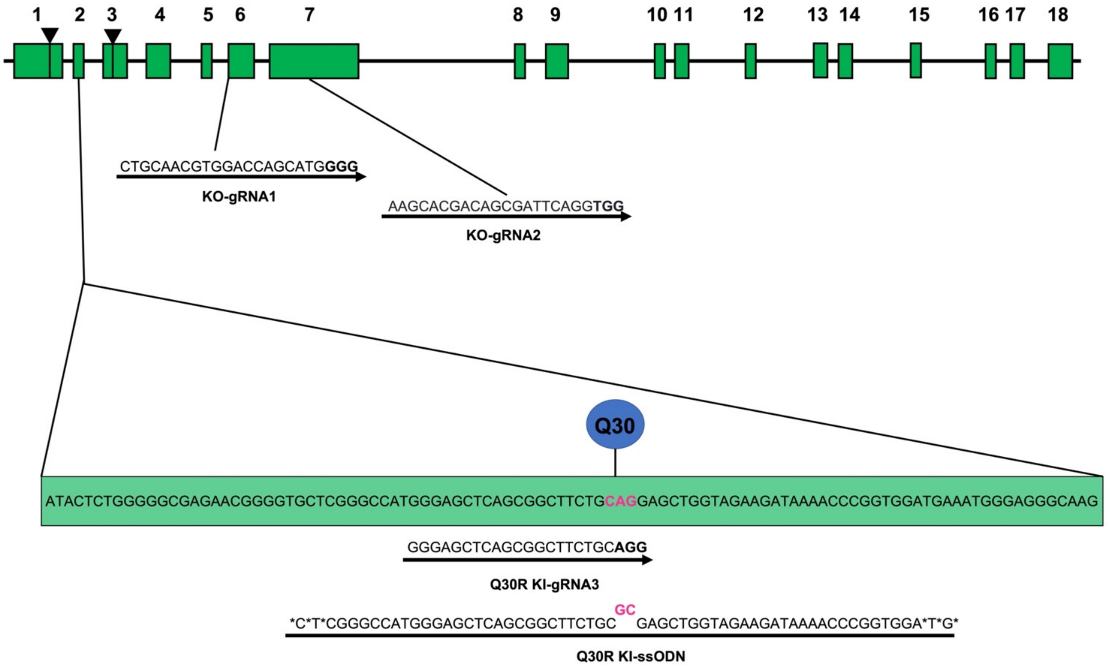

Figure S1: Pictorial representation of guide RNA and ssODN design for CRISPR/Cas9-mediated *AMBRA1* Q30R editing in hTERT-RPE1 cells.

KO—knockout; KI—knock-in; gRNA—guide RNA; ssODN—single-stranded oligodeoxynucleotide.

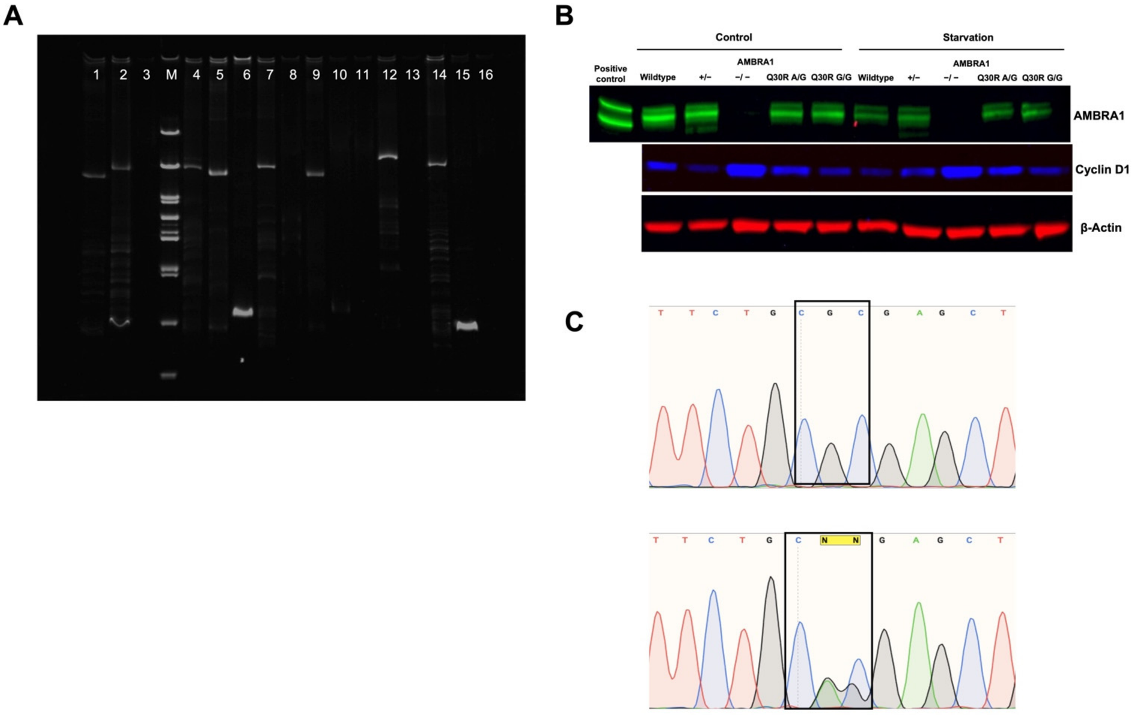

Figure S2: Confirmation of AMBRA1 knock-out and AMBRA1 Q30R editing of hTERT-RPE1 cells.

A. PAGE Analysis of *AMBRA1* knock-out. M – 100bp DNA ladder, Lane 6, 15 – Biallelic knock-outs showing 204bp, Lane 2 – Monoallelic knock-out with both 1300bp and 204 bp.

B. Western blot showing AMBRA1 (indicated as green bands) and cyclin D1 (indicated as blue bands) protein expression. AMBRA1 overexpression lysate purchased from Novus biologicals was used as positive control. β-actin was used as loading control.

C. Sanger sequencing chromatogram showing homozygous GG (AMBRA1$^{Q30R\ G/G}$) (top) and heterozygous AG (AMBRA1$^{Q30R\ A/G}$) (bottom) genotypes.

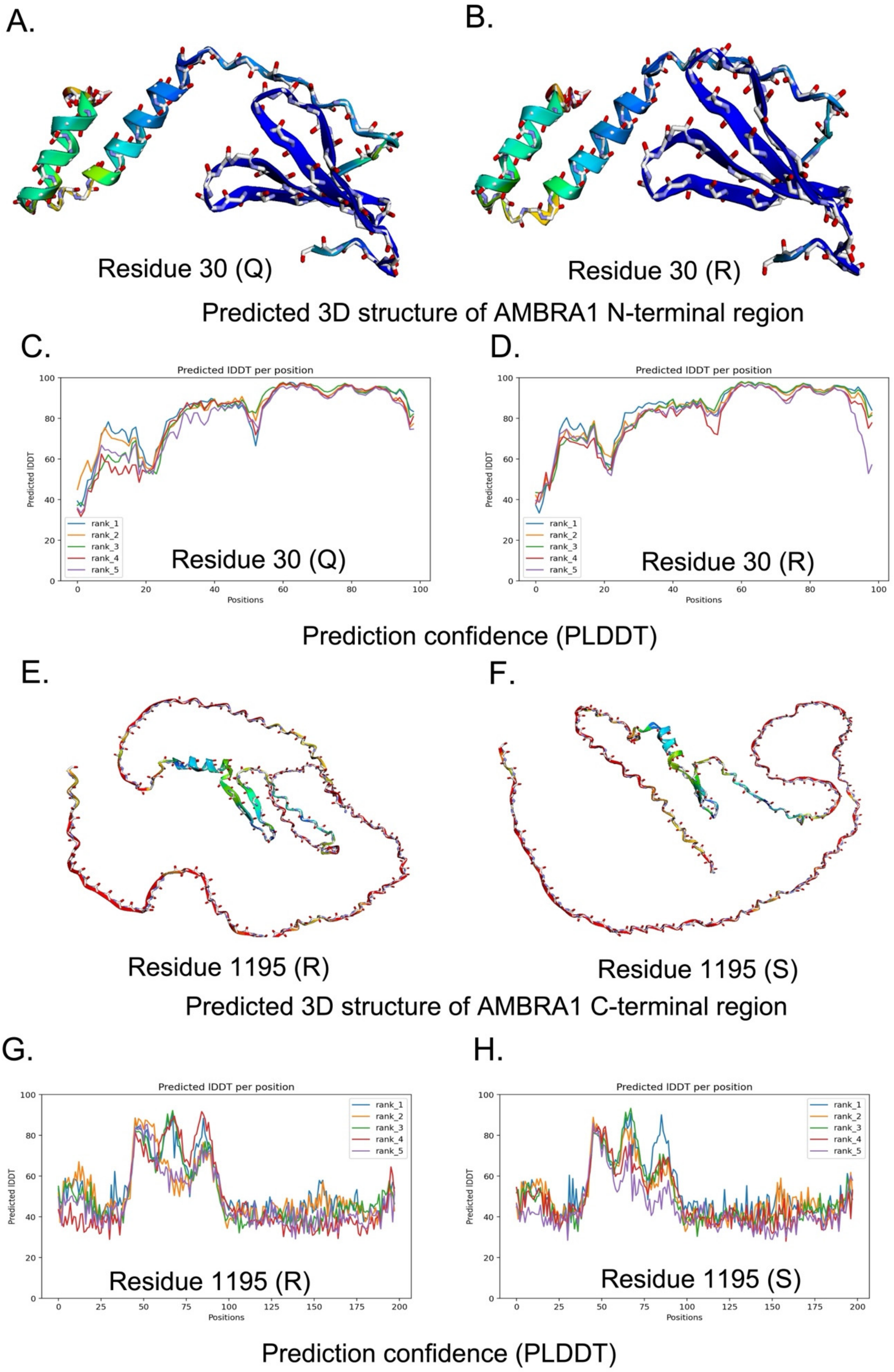

Figure S3: AlphaFold2-generated 3D protein structure for the wildtype and mutation residue of the AMBRA1 Q30R and R1195S mutations.

A,B. Predicted 3D structure of the AMBRA1 N-terminal region comprising Gln(Q) (wildtype) and Arg(R) (mutation) at position 30 and in the WD1 domain.

C,D. Prediction accuracy of AlphaFold2 as indicated by the pLDDT scores for the computed structures of the AMBRA1 N-terminal region with Q and R at position 30.

E,F. Predicted 3D structure of the AMBRA1 C-terminal region comprising Arg(R) (wildtype) and Ser (S) (mutation) at position 1195 and in the disordered region.

G,H. Prediction accuracy of AlphaFold2 as indicated by the pLDDT scores for the computed structures of the AMBRA1 C-terminal region with R and S at position 1195.
